# Supplementary material for: Effects of Interaction of Protein Hydrolysate and Arbuscular Mycorrhizal Fungi Effects on Citrus Growth and Expressions of Stress-Responsive Genes (Aquaporins and SOSs) under Salt Stress
Source: J Fungi (Basel). 2023 Sep 29;9(10):983. doi: 10.3390/jof9100983 (PMC10607954; doi:10.3390/jof9100983)
Supplement: Supplementary file 1 [file jof-09-00983-s001.zip › jof-2594145-supplementary.pdf]

Supplementary Material Table S1. Specific primer sequences of genes used for qRT-PCR.

**Gene names**	**Accession number**	**Primer sequnces(5’→3’)**
Actin	XM_006464503	F：CATCCCTCAGCACCTTCC
		R：CCAACCTTAGCACTTCTCC
CsPIP1	Cs_ont_5g048130.1	F：TGGCATGATCTTTGCGCTTG
		R：ACCATGTAGAAGACAGCCCTTG
CsPIP2	Cs_ont_6g015270.1	F：TTTTACAGGGCTGGCATTGC
		R：AAGATCATGCCGCCAAAAGC
CsPIP3	Cs_ont_6g020260.1	F：ATTGGGCGCTGAGATCATTG
		R：TTGACAATGGTGCCAAGACG
CsPIP4	Cs_ont_6g020270.1	F：TCGCCACGCTCTTGTTTTTG
		R：AATGCCAACACCACCACATC
CsPIP5	Cs_ont_6g020280.1	F：ATGTGGTGGTGTTGGCATTC
		R：AGAACAGCCCAAATGTCACC
CsPIP6	Cs_ont_7g001120.1	F：TCTTCGTGCTCGTTTACTGC
		R：AGAAAAGTGCACGTGTCAGC
CsPIP7	Cs_ont_7g001130.1	F：GCCGAGTTCATAGCCACTTTTC
		R：AAACGAGCGCGAAGATCATG
CsPIP8	Cs_ont_7g006190.1	F：TTGGCCACAATCCCAATCAC
		R：TGGTAGAAAGCCGCAATTGC
CsPIP9	Cs_ont_8g004900.1	F：TGGGTTTGCTGTGTTCATGG
		R：TCATCCCAGGCTTTCTCCTTG
CsPIP10	Cs_ont_8g028000.1	F：TATTGCATGGGCCTTTGGTG
		R：TTGCCAAGAACAGCCCAAAC
CsTIP1	Cs_ont_1g017160.1	F：TTTGTCGCTGTTGCAGTTGG
		R：AAGATGCCAGTGAGGATGGTG
CsTIP2	Cs_ont_4g004270.1	F：TGGCTCATGCTTTTGTGGTG
		R：GTGATGTGGCCACCGAAAAG
CsTIP3	Cs_ont_5g016210.1	F：TGTTGCCGGCAGAATTTCAC
		R：AATGCCAGTGGAAACCCAAC
CsTIP4	Cs_ont_5g027860.1	F：ATGCACACACCCAACAACAG
		R：AAAGCTGCTTCTGCTTCTGC
CsTIP5	Cs_ont_5g042380.1	F：TCGCCACGCTTCTTTTTGTG
		R：GCAACACCAACAAACAGTGC
CsTIP6	Cs_ont_6g022100.1	F：TTTGTCTTTGCCGGTGAAGG
		R：AGCAACGAAAAGGGCGAAAG
CsTIP7	Cs_ont_7g004240.1	F：TTGGAAACATCGGCATTCGC
		R：TTTGGATCCACTGCTGTTGC
CsTIP8	Cs_ont_8g003390.1	F：ATGCATTTGCCCTCTTCGTG
		R：AATGTAATGTGGCCGCCAAC
CsTIP9	Cs_ont_9g023550.1	F：TTGGCAATTGGGATGATGGC
		R：TTGAAACTTCCGGCAACCAC
CsSOS1	Cs_ont_2g014530.1	F：ACAAGGCAAGGCAACAGAAG
		R：AACGCCTTTGAACGTCAACC

CsSOS2	Cs_ont_4g006040.1	F：TTTGGCAAGCCGAACAAAGG
		R：TGACAATGAGCAACCGCATC
CsSOS3	Cs_ont_5g031770.1	F：TGACCCAGAAGAGTGGAAAGAG
		R：AAAGCTGGGAAATGCCAAGG
